# Prevention of perioperative venous thromboembolic complications using pneumatic compression cuffs in oral cancer patients in maxillofacial surgery

**DOI:** 10.1007/s00784-024-05987-7

**Published:** 2024-10-11

**Authors:** P. Römer, M. Krüger, B. Al-Nawas, P. W. Kämmerer, J. Heider

**Affiliations:** Department of Oral and Maxillofacial Surgery, Facial Plastic Surgery, University Medical Center Mainz, Augustusplatz 2, 55131 Mainz, Germany

**Keywords:** Thrombosis, Pulmonary embolism, Pneumatic compression, Oral squamous cell carcinoma, Oral and maxillofacial surgery

## Abstract

**Objectives:**

Venous thromboembolism (VTE) is still considered to be a significant medical issue. Physical measures to prevent perioperative venous thrombosis include early mobilization and intermittent pneumatic compression (IPC). The aim of this study was to evaluate whether IPC can reduce the incidence of postoperative thromboembolic events in patients with oral squamous cell carcinoma (OSCC) undergoing maxillofacial surgery.

**Materials and methods:**

Between March 2020 and May 2021, 75 patients with OSCC who did not receive perioperative prophylaxis using IPC were retrospectively examined to determine the occurrence of postoperative thromboembolism. Accordingly, 79 patients who received perioperative thrombosis prophylaxis using an IPC system as part of surgical tumor therapy from May 2021 to September 2023 were included in the study. The primary outcome measure was the occurrence of postoperative thromboembolism.

**Results:**

In the control group without IPC, thromboembolic events were observed in five out of 75 patients during postoperative hospitalization. In the intervention group, no thromboembolic occurrences were identified among the 79 patients studied (*p* = 0.02). The mean Caprini score in the control group was 7.72, whereas in the intervention group it averaged 8.30 (*p* = 0.027).

**Conclusions:**

The implementation of IPC-devices as supplementary perioperative thrombosis prophylaxis resulted in a notable decrease in postoperative venous thromboembolism (Number Needed to Treat = 15), which is why implementation of the system as a regular part of the clinical routine for perioperative management of OSCC patients can be considered a sensible approach.

**Clinical relevance:**

The use of IPC enhances patient outcomes and may lead to improved postoperative care protocols in this high-risk patient population.

## Introduction

Venous thromboembolism (VTE), including deep vein thrombosis (DVT), superficial vein thrombosis (SVT), and pulmonary embolism (PE) are considered a major medical and social problem. The annual incidence of venous thromboembolism (VTE) within the general populace approximates 100 instances per 100,000 individuals (0.1%), whereby approximately one-third of these cases manifest as symptomatic pulmonary embolism [1]. In non-oncological patients in the field of oral and maxillofacial surgery, an incidence of VTE between 0.15% and 2.8% is reported [2–6]. The frequency of venous thromboembolism and pulmonary embolism, however, is significantly increased in patients diagnosed with malignant tumors when compared to healthy individuals from the general population without underlying malignancies [7]. Depending on the entity, between 2 and 15% of tumor patients are affected by clinically apparent VTE [8, 9]. Oncological patients in oral and maxillofacial surgery commonly present several serious risk factors, including prolonged immobilization, active malignant tumor disease, nicotine and/or alcohol abuse, and advanced age, which significantly increases their risk of VTE or PE [10]. However, only limited studies are available to provide reliable data on the occurrence of thromboembolic events in oncological patients undergoing oral and maxillofacial surgery. In present studies involving head and neck tumor surgery, the reported incidence rates range from 0% for procedures with primary plastic wound closure and up to 6% for those with microvascular flap reconstruction [11–14].

Recent hospitalization, especially in conjunction with major surgery, is among one of the most important risk factors for the development of VTE in tumor patients [15–18].

Patients who do not receive adequate prophylaxis during their postoperative hospital stay are estimated to develop VTE in up to 20% and PE in an estimated 7% of cases, which, however, carry an increased risk of lethality [19–21]. Therefore, a thorough risk assessment of hospitalized patients according to their individual VTE risk and compliance with appropriate preventive measures during inpatient treatment is crucial to avoid thromboembolic complications [22, 23]. The Caprini Score is considered the best-validated individual risk assessment model for postoperative VTE [24–26]. Intermittent pneumatic compression (IPC) devices are used to prevent VTE by enhancing venous blood flow and reducing stasis. Sequentially inflating and deflating cuffs around the legs promote circulation and stimulate fibrinolysis, reducing the risk DVT and PE. The primary objective of this study was to determine whether the use of IPC reduces the incidence of postoperative thromboembolic events in patients who underwent tumor resection with and without microvascular reconstruction.

## Materials and methods

### Study cohort

A total of 154 patients (92 males and 62 females) were examined in this retrospective cohort study at the Department of Oral and Maxillofacial Surgery, Facial Plastic Surgery, University Medical Center Mainz, Germany. All participants consented in advance to the procedure and data collection. This study was approved by the local ethics committee of Rhineland-Palate, Mainz, Germany (registration number: 2024–17572) on June 5, 2024, and was conducted in accordance with the protocol and in compliance with the moral, ethical, and scientific principles governing clinical research set out in the Declaration of Helsinki of 1975 and revised in 1983. Between May 2021 and September 2023, 79 patients who received additional prophylaxis using Intermittent pneumatic compression were examined regarding postoperative thromboembolic events (test group). A control group of 75 patients who did not receive additional intermittent pneumatic compression device intervention between March 2020 and May 2021 was analyzed for comparison.

### Standard procedure for perioperative anticoagulation

Due to the a priori increased risk of thrombosis in patients with malignant tumors, all patients received anticoagulation with low-molecular-weight heparins (LMWH) on inpatient admission. Dalteparin (Fragmin p forte; PFIZER PHARMA GmbH, Berlin, Germany) was administered preoperatively at a dosage of 5,000 I.U. subcutaneously once daily. Depending on the surgical procedure performed, this regimen was maintained postoperatively or escalated to twice daily (morning and evening) for microvascular reconstruction. Patients who received a microvascular flap were also intraoperatively administered 5,000 I.U. heparin (heparin-sodium-5000-ratiopharm^®^; ratiopharm GmbH, Graf-Arco-Straße, Ulm, Germany) subcutaneously once after implementation of the arterial vascular anastomosis. The respective medications were continued in the postoperative inpatient phase until the patient was fully mobilized.

### Intermittent pneumatic compression (IPC)

The test group used a Kendall SCDTM Series 700 IPC system (Cardinal Health Germany 507 GmbH, Südportal, Norderstedt, Germany). The system consisted of a control device and variable-sized cuffs that are worn around the thighs and lower legs. Once the compression cuffs were fitted and the hose system was connected to the control unit, continuous intermittent pressure was applied automatically to the extremities with a pressure of 40 mmHg in the calf region and 30 mmHg in the thigh area at a respective duration of 11 s. This sequential compression provides unidirectional blood flow [27], reducing the risk of distal blood pooling [28] and maximize femoral blood flow velocity [29]. According to various clinical trials, a continuous circulation of a blood volume up to 7.8 L per hour can be achieved using IPC [30]. The frequency of the compression cycles is based on the patient’s venous refill time, which was automatically determined by the device. The cuffs were worn uninterruptedly from the onset of surgery until the patient achieved complete mobilization, with a minimum duration of two days postoperatively while the anticoagulation regimen prescribed above was maintained.

### Data acquisition and analyzed parameters

All data used for the analysis were obtained from digital patient files and anesthesia protocols from the Internal Hospital Information System (SAP Deutschland SE & Co. KG, Walldorf, Germany) at the University Medical Center Mainz. In addition to demographic factors such as age and sex, the parameters analyzed encompassed the patient’s medical history regarding prior thrombotic events and their routine use of anticoagulant medication. Furthermore, a distinction was made between the following procedure categories: (1) tumor resection solely with stage-dependent neck dissection together with local reconstruction and (2) resection with primary microvascular reconstruction within the control group (IPC-) and the test group (IPC+). Lastly, the patient records regarding postoperative thrombosis during the inpatient stay were reviewed. In the case of VTE or PE, the time of the event was further categorized as < 24 h postoperative, 24–48 h postoperative, and > 72 h postoperative.

### Statistics

The null hypothesis states that the frequency of postoperative thromboembolism does not differ between the groups with (IPC+) and without (IPC-) intermittent pneumatic leg compression. The primary outcome of the study was the occurrence of a postoperative venous embolism. The raw datasets obtained were entered into Excel^®^ spreadsheets (Microsoft Corporation, Redmond WA, USA) and imported into SPSS Statistics (version 27 MacOS X; SPSS Inc., IBM Corporation, Armonk, NY, USA). Descriptive statistics were used to present the data, with mean (m) ± standard deviation (SD±), minimum (min), and maximum (max) values reported. A non-parametric Shapiro-Wilk test was used to check for normal distribution. The data of the different groups were then analyzed for statistical significance depending on their variance and normal distribution using the unpaired Student’s t-test, Welch’s t-test, and Mann-Whitney U-Test. Histograms and bar charts were used for illustration purposes using GraphPad Prism (GraphPad Software, 225 Franklin Street. Fl. 26, Boston, MA 02110). To conduct the power analysis, effect sizes using Cohen’s h were calculated based on the proportions of the outcome of interest in both treatment and control groups. With this effect size, along with population sizes of each group and a significance level of 0.05, Python statsmodels 0.14.1 [31] was utilized to calculate the statistical power. The effect size calculated by Cohen’s d is 0.522, which results in a power of 89.97% with observed thrombosis rates of 0 and 6.67%.

## Results

A comprehensive analysis encompassed 154 patients with a mean age of 67 years (range 34–89 years), including 79 individuals subjected to additional intervention with intermittent pneumatic compression splints and 75 individuals receiving no supplementary treatment.

**Fig. 1:**
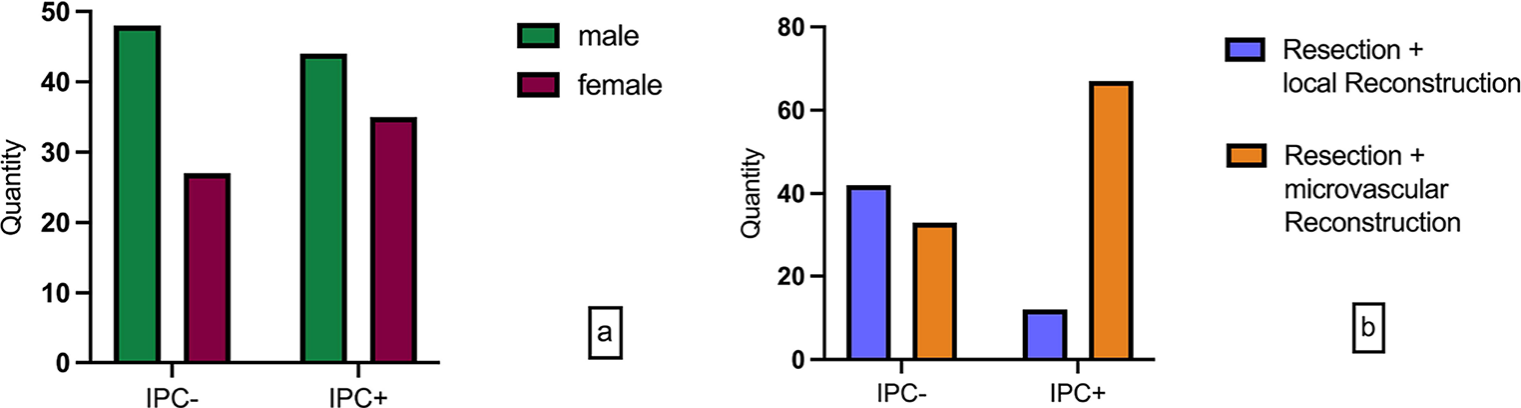
**a** study participants in the study groups by gender **b** surgical procedure in various groups

92 male and 62 female patients (*p* = 0.297) were examined. The IPC + intervention group consisted of 44 male and 35 female patients, while the IPC- group comprised 48 male and 27 female patients. Overall, 100 patients underwent surgical tumor resection with stage-dependent Neck Dissection and subsequent primary microvascular reconstruction (60 radial forearm flaps, 19 ulnar forearm flaps, 14 osteomyocutaneous fibula flaps, 4 ALT flaps, and 3 latissimus flaps). In comparison, 54 patients were treated with local reconstruction. The IPC + group accounted for 67 patients with microvascular reconstruction and 12 with primary local wound closure. In the control group IPC-, 42 patients underwent microvascular reconstruction, and 33 were treated with resection and local reconstruction (Fig. 1) (Table 1).

**Fig. 2:**
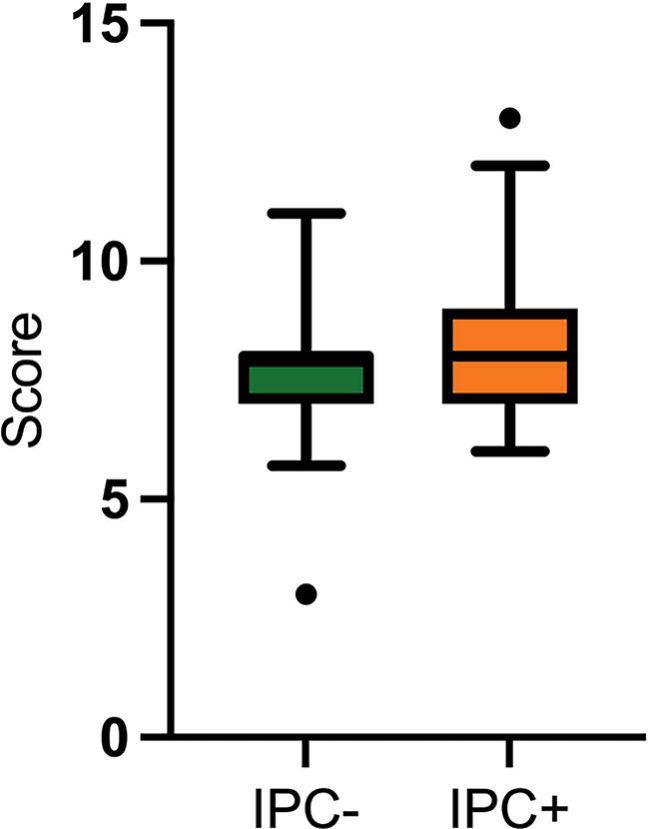
Caprini score achieved by the different study groups IPC- and IPC+

**Fig. 3:**
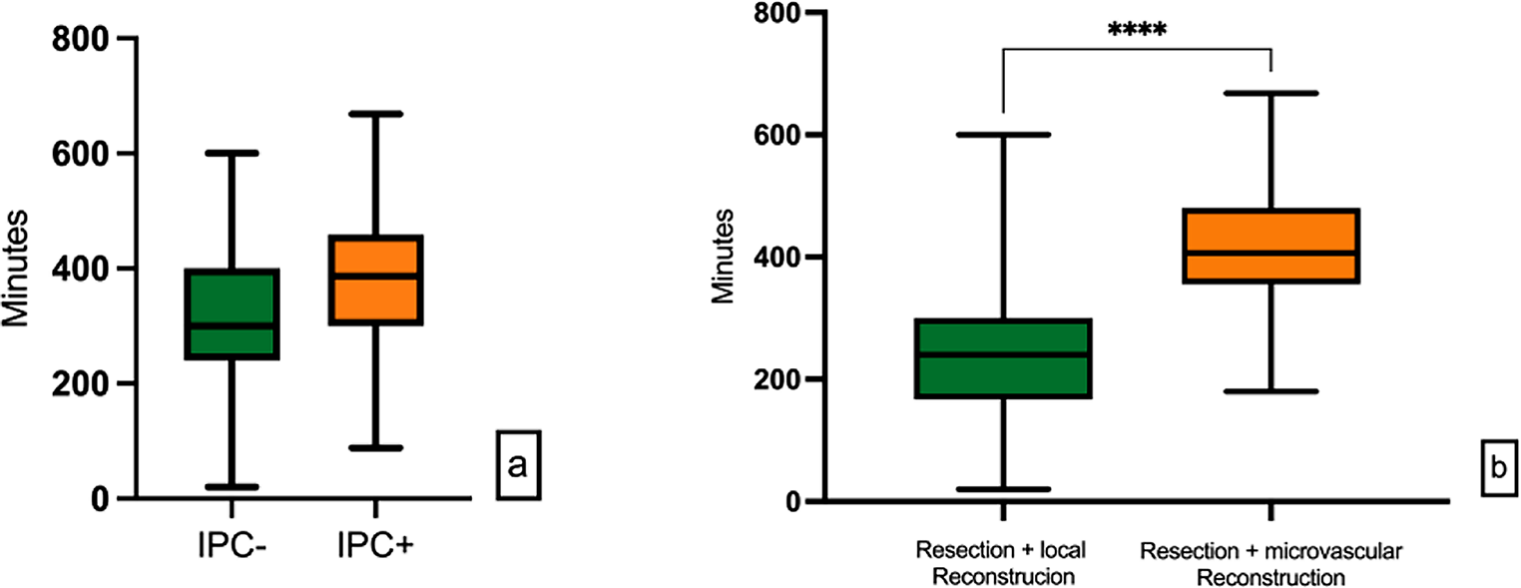
**a** surgery time of IPC- and IPC + group **b** surgery time depending on surgical intervention

The average surgery duration under intubation anesthesia was 351 min (20–668 min), in the case of microvascular reconstruction 414 min (180–668 min) and for tumor resection without microvascular graft 235 min (20–600 min) respectively (*p* < 0.001). Due to a substantially higher frequency of microvascular reconstructions in the IPC + cohort, the mean duration of surgical procedures exhibited a statistically significant increase compared to the IPC- control group (*p* < 0.001) (Fig. 2). Secondarily, the Caprini-Score [26] was used to assess the individual risk of thrombosis in each patient. On average, the study population scored 8.02 ± 1.47 (3–13). Specifically, the IPC- cohort attained a mean score of 7.72 ± 1.31 (3–11) points, whereas the IPC + intervention group achieved an average score of 8.30 ± 1.56 (6–13 points) (*p* = 0.027) (Fig. 3).

**Fig. 4:**
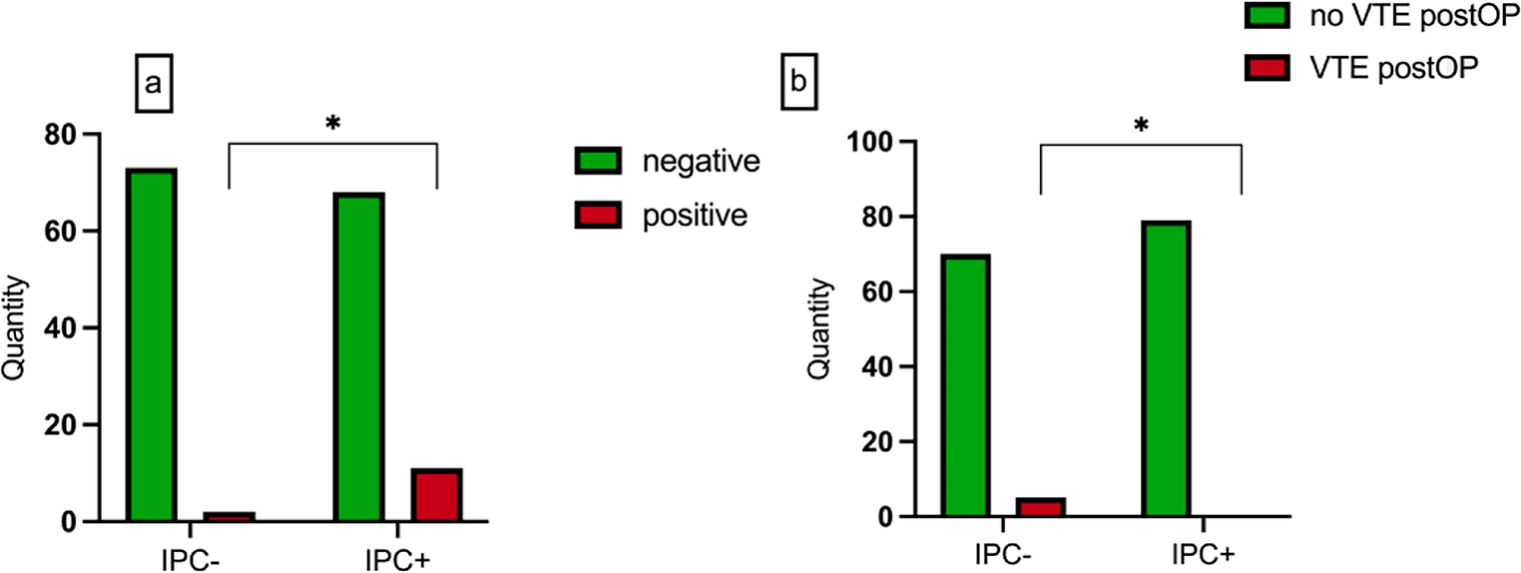
**a** patient cases with previous thromboembolic events in medical history **b** postoperative thromboembolic events by group

In 13 patients, thromboembolic events were identified in their medical history prior to surgery. Eleven of these patients with an increased individual risk of thrombosis were part of the IPC + intervention group (*p* = 0.012). During the postoperative inpatient stay, five patients developed a thromboembolic event resulting in a pulmonary embolism. All patients who experienced embolisms were part of the IPC- group. No thromboembolic complications were observed in the group of patients who received additional prophylaxis with intermittent pneumatic compression splints (*p* = 0.020) (Fig. 4).

**Fig. 5:**
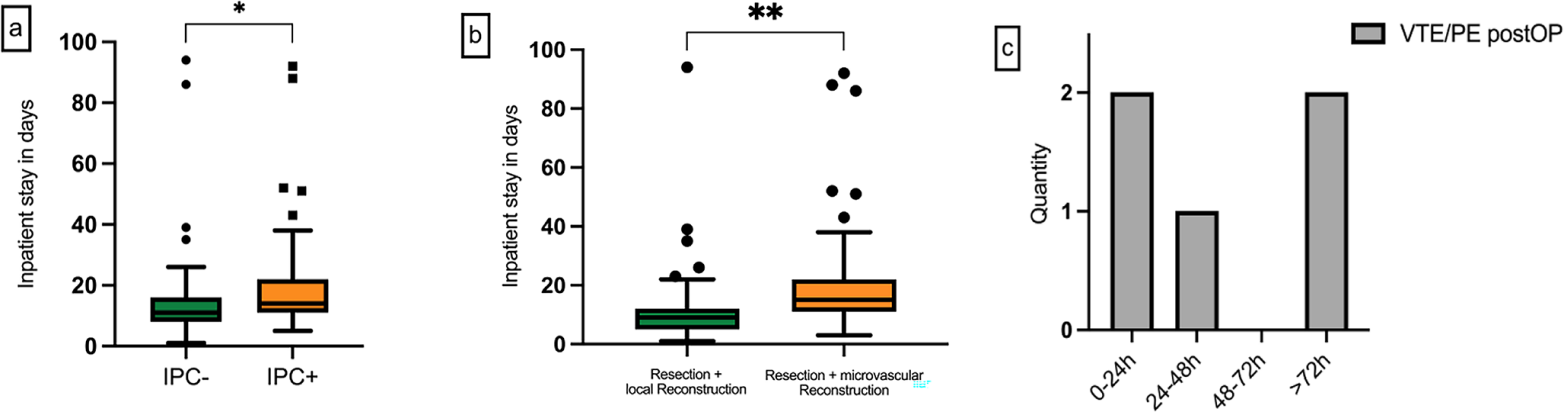
**a** average length of hospital stay per study cohort IPC- and IPC + **b** hospitalization by type of surgery performed; **c** time of postoperative thromboembolic events in the IPC- group

Among the five observed cases of pulmonary embolism, two incidents manifested within 24 h postoperatively, one event occurred between 24 and 48 h, and two transpired more than 72 h postoperatively (Fig. 5). The mean duration of hospitalization in the study population was 16.8 days. Patients who underwent tumor resection and local reconstruction showed a significantly shorter average hospitalization time (11.9 days) than patients who received simultaneous microvascular reconstruction (19.4 days) (*p* = 0.035). Consequently, the IPC + cohort also exhibited a significantly extended mean duration of hospitalization due to the aforementioned variances within the study population (IPC- 14.2 days; IPC + 19.3 days; *p* = 0.035) (Fig. 5).

With a total of five postoperative thrombotic events observed in the control group IPC-, corresponding to an incidence rate of 6.67%, a specific number needed to treat of 15 individuals can be determined.

**Table 1:** Overview of the parameters assessed for the two study groups

	IPC-^1^	IPC+^2^	Total	SD±^3^	*p*-value
Male	48	44	92		
Female	27	35	62		
Total	75	79	154		
T-Stadium					
T1	19	21	40		
T2	27	20	47		
T3	10	19	29		
T4	19	19	38		
Tumor resection + local reconstruction	42	12	54		
Tumor resection + microvascular reconstruction	33	67	100		
Mean surgery duration	307 min	392 min	351 min		< 0.001
local reconstruction + microvascular reconstruction			235 min 413 min	105 min 93 min	
No previous VTE^4^	73	68	141		
Previous VTE	2	11	13		0.012
VTE postoperative	5	0	5		0.020
local wound closure +microvascular reconstruction	2 3	0 0	2 3		0.649
T1	0	0	0		
T2	1	0	1		
T3	2	0	2		
T4	2	0	2		
Hospitalization (in days)	14.24	19.28	16.8		0.035
local wound closure			11.98 days	13.54 days	
+ microvascular reconstruction			19.44 days	15 days	
Caprini Score	7.72	8.3	8.02		0.027

1. Group with perioperative intermittent pneumatic compression

2. Group without perioperative intermittent pneumatic compression

3. Standard deviation

4. Venous thromboembolism

### Group analyses

The Pearson Chi-Square test and Fisher’s exact test were used to analyze the association between the type of surgery and the occurrence of postoperative thrombosis. The Pearson Chi-Square test showed no significant association (*p* = 0.456), which was confirmed by Fisher’s exact test (two-sided p-value = 0.649), indicating that the type of surgical procedure (with or without microvascular reconstruction) had no significant effect on the incidence of postoperative thrombosis.

## Discussion

The general risk of perioperative venous thromboembolism in oral and maxillofacial surgery is estimated to range between 0.15 and 1.6% [3, 4, 10, 32]. However, no studies are currently available regarding the use of IPC and its effectiveness in preventing VTE in oncological patients undergoing oral and maxillofacial surgery. Malignant diseases, trauma, major surgery, and previous thrombosis events have already been identified as predisposing risk factors in oral and maxillofacial surgery for a long time [33]. Thus, a recent study by Wang et al. reported an incidence of postoperative VTE of 20.7% in a cohort of 372 oncological patients who underwent tumor resection with simultaneous reconstruction [34]. Similar results were reported in a retrospective study by Kakei et al. from 2016, in which the incidence of sonographically detected DVT was 24%, whereas pulmonary embolisms only occurred in 2.3% of cases [35]. General measures to prevent postoperative VTE include discontinuing coagulative medication and early postoperative mobilization and individual risk stratification, for example, using the standardized Caprini Score [26, 34, 36]. This score classifies patients into specific risk profiles based on a point value achieved according to their predisposing factors. Studies have demonstrated a significant increase in the risk of DVT among patients categorized with scores ranging from nine to eleven, corresponding to an 18.7-fold increase, and those categorized with scores from 12 and higher, indicating a substantially heightened risk of 98.4 times compared to patients with allocated scores of five to eight [36]. However, it is imperative to acknowledge that patients undergoing surgical oncological interventions in oral and maxillofacial surgery inherently accrue a Caprini-Score of 4–6 due to their underlying disease, the prolonged duration of surgical procedures, and the anticipated temporary immobility after treatment, thus ranking them within the high-risk category for postoperative VTE. In cases where patients have a medical history of VTE or have familial predispositions, the score increases by an additional three points.

Consequently, apart from very few young patients (under 41 years of age) with no other comorbidities and a short surgery duration of less than 45 min, all patients in our study population scored 6 or higher. Current evidence from recent studies in head and neck surgery also indicates that intraoperative and postoperative application of LWMH can lead to fewer postoperative VTE cases and flap losses [37]. This recommendation concurs with the latest ASCO guideline on prophylaxis of VTE, according to which pharmacological thrombosis prophylaxis with LMWH should be continued for at least 7–10 days in patients with tumor diseases who undergo major cancer surgery [38, 39]. Adequate patient positioning and mechanical therapy, including intermittent pneumatic compression, represent additional strategies for preventing perioperative thrombosis. IPC is frequently employed as part of multimodal thromboprophylaxis strategies, particularly in orthopedic and abdominal surgeries, where patients face heightened VTE susceptibility, but also as an adjunctive treatment for immobilized patients in intensive care units and geriatric facilities among patients with reduced mobility and increased risk factors for VTE [40, 41]. Several studies have demonstrated reduced VTE using intermittent pneumatic compression in surgical and non-surgical patients [42, 43].

Furthermore, scientific literature has substantiated the positive effects on blood flow dynamics and fibrinolytic activity [27, 44–46]. Additional benefits of IPC include its user-friendly operation, reduced incidence of postoperative bleeding events, and synergistic potential when combined with other prophylactic modalities [42]. However, a comparative study showed that IPC alone is insufficient for perioperative VTE prophylaxis in high-risk patients with malignant diseases, and an intensified combination with pharmacological anticoagulation is recommended [42, 47, 48]. These findings align with a recent Cochrane review, indicating that the combined approach of IPC with pharmacological prophylaxis could substantially decrease the occurrence of postoperative PE and DVT [49]. Like numerous other publications, our study successfully demonstrated a significantly reduced incidence of postoperative thromboembolic events in patients receiving adjunctive prophylactic intermittent pneumatic compression therapy [50]. The study’s statistical power was calculated to be 89.97% at a 5% significance level, demonstrating a high probability of detecting true differences in thrombosis rates between the groups. The results also indicate no significant differences in the incidence of postoperative thrombosis between patients who underwent microvascular reconstruction and those who received local reconstruction. Potential limitations of the study include the heterogeneous patient population with different risk profiles and surgical procedures, as well as the varying duration of surgery and inpatient hospitalization.

## Conclusion

Combined thromboprophylaxis with IPC and LMWH resulted in a significant reduction of postoperative thromboembolism in patients with oral squamous cell carcinoma undergoing oncological surgery and reconstruction. In contrast to the cohort receiving conventional pharmacological prophylaxis with LMWH alone, exhibiting an incidence of 6.67% for postoperative VTE, no occurrences of VTE were observed in the group treated with additional IPC during the postoperative inpatient period. Based on these results, IPC technique was integrated into the clinical routine of our institution for patients undergoing major tumors or reconstructive surgery.

## Data Availability

Data that supports the findings of this study are available on reasonable request.

## Abbreviations

VTE: Venous thromboembolism
DVT: Deep vein thrombosis
PE: Pulmonary Embolism
IPC: Intermittent pneumatic compression
OSCC: Oral squamous cell carcinoma
LMWH: Low-molecular-weight heparin
IPC +: Intervention group with perioperative intermittent pneumatic compression
IPC-: Control group without perioperative intermittent pneumatic compression
M: Mean
SD ±: Standard deviation
Min: Minimum
Max: Maximum

## References

[1] Blann AD, Lip GY (2006) Venous Thromboembolism BMJ 332(7535):215–219 16439400 10.1136/bmj.332.7535.215 PMC1352055

[2] Van de Perre JP et al (1996) Perioperative morbidity in maxillofacial orthopaedic surgery: a retrospective study. J Craniomaxillofac Surg 24(5):263–270 8938506 10.1016/s1010-5182(96)80056-4

[3] Blackburn TK, Pritchard K, Richardson D (2006) Symptomatic venous thromboembolism after orthognathic operations: an audit. Br J Oral Maxillofac Surg 44(5):389–392 16213069 10.1016/j.bjoms.2005.08.008

[4] Forouzanfar T et al (2010) Incidence of venous thromboembolism in oral and maxillofacial surgery: a retrospective analysis. Int J Oral Maxillofac Surg 39(3):256–259 20018490 10.1016/j.ijom.2009.10.024

[5] Skorpil N et al (2012) Is thromboembolism prophylaxis necessary for low and moderate risk patients in maxillofacial trauma? A retrospective analysis. Int J Oral Maxillofac Surg 41(8):902–905 22321617 10.1016/j.ijom.2012.01.001

[6] Li CX et al (2023) Risk assessment of venous thromboembolism in head and neck cancer patients and its establishment of a prediction model. Head Neck 45(10):2515–2524 37548087 10.1002/hed.27475

[7] Lip GY, Chin BS, Blann AD (2002) Cancer and the prothrombotic state. Lancet Oncol 3(1):27–34 11908507 10.1016/s1470-2045(01)00619-2

[8] Johnson MJ, Sproule MW, Paul J (1999) The prevalence and associated variables of deep venous thrombosis in patients with advanced cancer. Clin Oncol (R Coll Radiol) 11(2):105–110 10378636 10.1053/clon.1999.9023

[9] Stein PD et al (2006) Incidence of venous thromboembolism in patients hospitalized with cancer. Am J Med 119(1):60–68 16431186 10.1016/j.amjmed.2005.06.058

[10] Williams B, Indresano AT, O’Ryan F (2011) Venous thromboembolism in oral and maxillofacial surgery: a review of the literature. J Oral Maxillofac Surg 69(3):840–844 21255893 10.1016/j.joms.2010.11.025

[11] Thai L et al (2013) Venous thromboembolism in patients with head and neck cancer after surgery. Head Neck 35(1):4–9 22302625 10.1002/hed.22920

[12] Gavriel H et al (2013) Safety of thromboprophylaxis after oncologic head and neck surgery. Study of 1018 patients. Head Neck 35(10):1410–1414 23169262 10.1002/hed.23158

[13] Chen CM et al (2008) The incidence of venous thromboembolism after oncologic head and neck reconstruction. Ann Plast Surg 60(5):476–479 18434817 10.1097/SAP.0b013e31816fd7e7

[14] Clayburgh D et al (2013) Prospective study of venous thromboembolism in patients with head and neck cancer after surgery: interim analysis. JAMA Otolaryngol Head Neck Surg 139(2):161–167 23429947 10.1001/jamaoto.2013.1372

[15] Heit JA et al (2001) Incidence of venous thromboembolism in hospitalized patients vs community residents. Mayo Clin Proc 76(11):1102–1110 11702898 10.4065/76.11.1102

[16] Amin AN et al (2012) Duration of venous thromboembolism risk across a continuum in medically ill hospitalized patients. J Hosp Med 7(3):231–238 22190427 10.1002/jhm.1002

[17] Stein PD, Beemath A, Olson RE (2005) Trends in the incidence of pulmonary embolism and deep venous thrombosis in hospitalized patients. Am J Cardiol 95(12):1525–1526 15950590 10.1016/j.amjcard.2005.02.030

[18] Zhan C, Miller MR (2003) Excess length of stay, charges, and mortality attributable to medical injuries during hospitalization. JAMA 290(14):1868–1874 14532315 10.1001/jama.290.14.1868

[19] Geerts WH et al (2001) Prevention of venous thromboembolism. Chest 119(1 Suppl):132S–175S 11157647 10.1378/chest.119.1_suppl.132s

[20] Mismetti P et al (2005) Enoxaparin in the treatment of deep vein thrombosis with or without pulmonary embolism: an individual patient data meta-analysis. Chest 128(4):2203–2210 16236875 10.1378/chest.128.4.2203

[21] Tooher R et al (2005) A systematic review of strategies to improve prophylaxis for venous thromboembolism in hospitals. Ann Surg 241(3):397–415 15729062 10.1097/01.sla.0000154120.96169.99 PMC1356978

[22] Nicolaides AN et al (2013) Prevention and treatment of venous thromboembolism–international Consensus Statement. Int Angiol 32(2):111–260 24402349

[23] Guyatt GH et al (2012) *Executive summary: Antithrombotic Therapy and Prevention of Thrombosis, 9th ed: American College of Chest Physicians Evidence-Based Clinical Practice Guidelines.* Chest, 141(2 Suppl): p. 7S-47S 10.1378/chest.1412S3 PMC3278060 22315257

[24] Pannucci CJ et al (2017) Individualized venous thromboembolism risk stratification using the 2005 Caprini score to identify the benefits and Harms of Chemoprophylaxis in Surgical patients: a Meta-analysis. Ann Surg 265(6):1094–1103 28106607 10.1097/SLA.0000000000002126

[25] Hanh BM et al (2019) Determination of risk factors for venous thromboembolism by an adapted Caprini Scoring System in Surgical patients. J Pers Med, 9(3) 10.3390/jpm9030036 PMC6789529 31319527

[26] Caprini JA et al (1991) Clinical assessment of venous thromboembolic risk in surgical patients. Semin Thromb Hemost 17(Suppl 3):304–312 1754886

[27] Kakkos SK et al (2005) Comparison of two intermittent pneumatic compression systems. A hemodynamic study. Int Angiol 24(4):330–335 16355089

[28] Abu-Own A, Scurr JH, Smith PDC (1993) Assessment of Intermittent Pneumatic Compression by Strain-Gauge Plethysmography. Phlebology 8(2):68–71

[29] Nicolaides AN, Fernandes e J, Fernandes, Pollock AV (1980) Intermittent sequential pneumatic compression of the legs in the prevention of venous stasis and postoperative deep venous thrombosis. Surgery 87(1):69–76 6985760

[30] Griffin M et al (2007) Comparison of three intermittent pneumatic compression systems in patients with varicose veins: a hemodynamic study. Int Angiol 26(2):158–164 17489080

[31] Seabold S, Perktold J (2010) *statsmodels: Econometric and statistical modeling with python*, in *9th Python in Science Conference*

[32] Lodders JN et al (2015) Incidence of symptomatic venous thromboembolism in oncological oral and maxillofacial operations: retrospective analysis. Br J Oral Maxillofac Surg 53(3):244–250 25640701 10.1016/j.bjoms.2014.12.001

[33] Lowry JC (1995) Thromboembolic disease and thromboprophylaxis in oral and maxillofacial surgery: experience and practice. Br J Oral Maxillofac Surg 33(2):101–106 7772581 10.1016/0266-4356(95)90209-0

[34] Wang X et al (2024) Development and validation of a nomogram for identifying venous thromboembolism following oral and maxillofacial oncological surgery with simultaneous reconstruction. Eur J Surg Oncol 50(1):107307 38048726 10.1016/j.ejso.2023.107307

[35] Kakei Y et al (2016) Incidence of venous thromboembolism after oral oncologic surgery with Simultaneous Reconstruction. J Oral Maxillofac Surg 74(1):212–217 26342948 10.1016/j.joms.2015.08.006

[36] Lobastov K et al (2016) Validation of the Caprini risk assessment model for venous thromboembolism in high-risk surgical patients in the background of standard prophylaxis. J Vasc Surg Venous Lymphat Disord 4(2):153–160 26993860 10.1016/j.jvsv.2015.09.004

[37] Grill FD et al (2024) Perioperative anticoagulation in head and neck free flap reconstructions: experience of an anticoagulative scheme and its modification. Microsurgery 44(1):e31096 37602929 10.1002/micr.31096

[38] Stockler MR (2020) ASCO updated recommendations for preventing and treating VTE in adults with cancer. Ann Intern Med 172(2):JC2 31958816 10.7326/ACPJ202001210-002

[39] Key NS et al (2020) Venous thromboembolism prophylaxis and treatment in patients with Cancer: ASCO Clinical Practice Guideline Update. J Clin Oncol 38(5):496–520 31381464 10.1200/JCO.19.01461

[40] Geerts WH et al (2008) *Prevention of venous thromboembolism: American College of Chest Physicians Evidence-Based Clinical Practice Guidelines (8th Edition).* Chest, 133(6 Suppl): p. 381S-453S 10.1378/chest.08-0656 18574271

[41] Kahn SR et al (2012) Prevention of VTE in nonsurgical patients: Antithrombotic Therapy and Prevention of Thrombosis, 9th ed: American College of Chest Physicians evidence-based clinical practice guidelines. Chest, 141(2 Suppl): p. e195S-e226S. 10.1378/chest.11-2296 PMC3278052 22315261

[42] Ho KM, Tan JA (2013) Stratified meta-analysis of intermittent pneumatic compression of the lower limbs to prevent venous thromboembolism in hospitalized patients. Circulation 128(9):1003–1020 23852609 10.1161/CIRCULATIONAHA.113.002690

[43] Urbankova J et al (2005) Intermittent pneumatic compression and deep vein thrombosis prevention. Thromb Haemost 94(12):1181–1185 16411391 10.1160/TH05-04-0222

[44] Weitz J et al (1986) Effects of intermittent pneumatic calf compression on postoperative thrombin and plasmin activity. Thromb Haemost 56(05):198–201 2949390

[45] Lurie F et al (2008) On the mechanism of action of pneumatic compression devices: combined magnetic resonance imaging and duplex ultrasound investigation. J Vasc Surg 48(4):1000–1006 18572366 10.1016/j.jvs.2008.04.009

[46] Jacobs DG et al (1996) Hemodynamic and fibrinolytic consequences of intermittent pneumatic compression: preliminary results. J Trauma, 40(5): p. 710 – 16; discussion 716-7. 10.1097/00005373-199605000-00005 8614068

[47] Clarke-Pearson DL et al (2003) Venous thromboembolism prophylaxis: patients at high risk to fail intermittent pneumatic compression. Obstet Gynecol 101(1):157–163 12517661 10.1016/s0029-7844(02)02444-4

[48] Silbersack Y et al (2004) Prevention of deep-vein thrombosis after total hip and knee replacement: low-molecular-weight heparin in combination with intermittent pneumatic compression. J Bone Joint Surg Br Volume 86(6):809–812 10.1302/0301-620x.86b6.13958 15330019

[49] Kakkos S et al (2022) Combined intermittent pneumatic leg compression and pharmacological prophylaxis for prevention of venous thromboembolism. Cochrane Database Syst Rev 1(1):CD005258 35089599 10.1002/14651858.CD005258.pub4 PMC8796751

[50] Parry K et al (2017) Intermittent pneumatic compression in combination with low-molecular weight heparin in the prevention of venous thromboembolic events in esophageal cancer surgery. J Surg Oncol 115(2):181–185 28054341 10.1002/jso.24480

